# Repetitive Transcranial Magnetic Stimulation for Refractory, Widespread Complex Regional Pain Syndrome Type II: A Case Report

**DOI:** 10.7759/cureus.115352

**Published:** 2026-08-28

**Authors:** Jared S Garland, Christopher J Kim, Christina La Croix, Xiaoning Yuan, Paul F Pasquina

**Affiliations:** 1 Neurology, Barnes-Jewish Hospital, St. Louis, USA; 2 Physical Medicine and Rehabilitation, Lewis Katz School of Medicine of Temple University, Philadelphia, USA; 3 Psychiatry/Physical Medicine and Rehabilitation, Uniformed Services University of the Health Sciences, Bethesda, USA; 4 Physical Medicine and Rehabilitation, Uniformed Services University of the Health Sciences, Bethesda, USA; 5 Physical Medicine and Rehabilitation, Walter Reed National Military Medical Center, Bethesda, USA

**Keywords:** ankle sprain, case report, complex regional pain syndrome, military, motor cortex, neuromodulation, neuropathic pain, repetitive transcranial magnetic stimulation

## Abstract

Complex regional pain syndrome (CRPS) is a poorly understood pain disorder often resistant to conventional therapies. Although typically localized, CRPS can spread to other regions. Repetitive transcranial magnetic stimulation (rTMS) is a non-invasive treatment showing promise for neuropathic pain associated with CRPS.

A 33-year-old active-duty service member developed widespread, refractory CRPS following a severe ankle sprain, with persistent pain and functional limitations despite medications, ketamine infusions, and sympathetic nerve blocks. The patient received 21 rTMS sessions over four weeks, targeting the left primary motor cortex. Pain scores decreased from 5.9 to 3.8 (legs) and 5.3 to 3.3 (hands) following treatment sessions. A 10% pain reduction was sustained at one-month post-treatment. No significant adverse effects were observed.

rTMS was safe, well-tolerated, and associated with clinically meaningful pain reduction in this case of refractory, widespread CRPS, supporting further investigation of rTMS for severe CRPS.

## Introduction

Complex regional pain syndrome (CRPS) is a challenging pain disorder, often characterized by persistent regional pain, allodynia, and hyperalgesia that is disproportionate to the underlying injury and refractory to standard treatments [1,2]. The disorder is classified as Type I (without identifiable nerve injury) or Type II (with confirmed nerve damage). The Budapest criteria for CRPS require ongoing pain disproportionate to the inciting trauma, reported symptoms in at least three clinical categories (sensory, vasomotor, sudomotor/edema, motor/trophic) with objective signs observed in at least two, and the exclusion of any alternative diagnosis [3].

CRPS is a rare condition with a five-year incidence of 0.07% in the United States [3]. Although acute symptoms often improve rapidly within the first six months, 14-27% of patients continue to fulfill diagnostic criteria for CRPS at 12 months [3]. While typically confined to a single limb, symptom spread occurs in approximately 20% of patients, most commonly contralaterally (63%), followed by ipsilaterally (34%) and diagonally (3%) [4]. Patients with multi-limb CRPS tend to have a longer disease duration and are more prone to movement and behavioral health symptoms, including depression and anxiety [4]. This marked heterogeneity complicates management and underscores the critical need for novel therapeutic strategies.

Repetitive transcranial magnetic stimulation (rTMS) is a noninvasive, safe, and well-tolerated neuromodulation intervention that is approved by the Food and Drug Administration for major depressive disorder, obsessive-compulsive disorder, and migraine [5]. rTMS employs magnetic pulses to modulate cortical excitability, influencing neuroplasticity and inducing long-lasting changes in both the targeted cortical area and connected regions via trans-synaptic pathways [3].

Previous research has demonstrated rTMS efficacy in various chronic pain conditions, including neuropathic pain. A multidisciplinary panel from the International Neuromodulation Society rated the overall level of evidence supporting rTMS for neuropathic pain as high, recommending further exploration of its therapeutic potential [6]. Specifically, stimulation of the primary motor cortex (M1) is believed to provide top-down modulation of pain perception and has been shown in open-label studies to produce significant pain reduction in CRPS patients without serious adverse effects [6,7]. High-frequency primary motor cortex rTMS is hypothesized to attenuate CRPS and neuropathic pain by restoring GABAergic intracortical inhibition, driving descending endogenous opioid pathways, and regulating central neuroinflammatory signaling [8,9]. Studies also suggest that combining rTMS with physical therapy and analgesics leads to statistically significant pain reduction compared with pharmacologic therapy alone [7,10].

Based on these findings, rTMS represents a promising adjunctive therapy for CRPS. The aim of this case study is to document the clinical outcomes following a course of rTMS in a patient with multi-limb, treatment-resistant CRPS Type II refractory to standard-of-care interventions.

## Case presentation

This case study was reviewed and received an exempt determination by the local Institutional Review Board. The patient was fully informed of the potential risks and benefits and provided written informed consent for this case report.

The patient, a previously healthy 33-year-old male military officer, sustained a severe right ankle sprain while stationed overseas. After surgical treatment for a displaced right talar dome fracture and a subsequent arthroscopic repair for a non-healing osteochondral defect, the patient developed characteristic CRPS symptoms, including vasomotor changes, allodynia, and severe, constant pain. The symptoms subsequently spread contralaterally to involve the left foot and ankle.

Over the 22 months from CRPS diagnosis to rTMS initiation, a variety of conservative and invasive interventions failed to provide meaningful, sustained pain relief. These included debridement, a popliteal nerve block, a cartilage matrix allograft, transcutaneous nerve stimulation, acupuncture, and ketamine infusions. CRPS symptoms progressively spread to involve the hands and left leg. Three lumbar sympathetic nerve blocks provided only transient relief, and full therapeutic trials of multiple pharmacologic agents, including oxycodone, tramadol, pregabalin, gabapentin, nortriptyline, amitriptyline, celecoxib, dronabinol, eszopiclone, zolpidem, sertraline, tapentadol, ondansetron, and various nonsteroidal anti-inflammatory drugs (NSAIDs), did not result in sustained clinical benefit.

Following these multiple unsuccessful interventions, the patient was referred for rTMS therapy. The patient’s evaluation, protocol selection, and course of rTMS therapy were led and supervised by a Physical Medicine and Rehabilitation physician. At the initiation of treatment, his active medication regimen included tapentadol, ondansetron, zolpidem, and NSAIDs. The patient continued both physical therapy and analgesic medications throughout the rTMS course, with medication reconciliation and safety assessments conducted prior to each treatment session. Physical therapy (2-3 times a week) included range-of-motion exercises, tactile desensitization, graded motor imagery, and modalities (e.g., heat, fluidotherapy, transcutaneous electrical nerve stimulation) to facilitate an active program, with progressive functional loading, aerobic conditioning, and work-specific training.

Assessments

To track clinical progression, pain intensity and overall health status were evaluated using validated outcome measures. Pain intensity was measured using the Defense and Veterans Pain Rating Scale (DVPRS), an instrument with established internal consistency, test-retest reliability, and construct validity in military populations [11]. Overall health status was assessed using the United States Department of Health and Human Services' Centers for Disease Control and Prevention Health-Related Quality of Life-14 (HRQOL-14), a validated 14-item instrument with established test-retest reliability and construct validity for monitoring physical and mental health trajectories, functional impairment, and quality of life in patients with complex, chronic medical conditions [12].

Baseline assessments included the DVPRS and HRQOL-14. During the treatment phase, the DVPRS was administered daily before each rTMS session. The Visual Analog Scale (VAS) component of the DVPRS, which captured current pain, was also completed immediately post-session. Treatment tolerability and safety were monitored daily following each rTMS session using a structured side-effect tracking log based on established rTMS safety monitoring guidelines [13,14]. This checklist recorded the presence, severity, and duration of common treatment-emergent adverse events, such as localized scalp discomfort, headache, facial muscle twitching, or neck stiffness [13,14].

rTMS procedure

All rTMS sessions were delivered using the NeuroStar Advanced Therapy System (Neuronetics, Malvern, PA). The resting motor threshold (rMT) was determined by stimulating the left primary motor cortex (M1) with an adaptive staircase method, observing the contralateral abductor pollicis brevis (APB) muscle response. The rMT was defined as the minimum stimulation intensity that elicited a visible APB contraction in 50% of trials.

The M1 cortical representation site for the contralateral leg was identified by rotating the stimulation coil upward 25° from the M1 APB representation site, and the site for the contralateral hand was identified by rotating the coil upward 2° from the same reference point.

A total of 21 rTMS sessions were administered daily (Monday to Friday) over four weeks. All stimulation was delivered at 80% of the patient’s rMT, with the procedure adjusted over the treatment course. Sessions 1-7 targeted the M1 leg site contralateral to the patient’s area of greatest pain (left leg). Each session consisted of 50 trains of five-second stimulation at 10 Hz, with a 25-second inter-train interval (2,500 pulses, 25 minutes/session). Sessions 8-21 targeted both the M1 leg and M1 hand sites to address left leg and hand pain. Each session included 40 trains of five-second stimulation at 10 Hz delivered to the M1 leg site, with a 25-second inter-train interval, followed by 40 trains delivered to the M1 hand site, with a 25-second inter-train interval (4,000 pulses, 40 minutes/session). The selection of high-frequency (10 Hz) rTMS targeting M1 at 80% rMT across 21 sessions was derived from evidence-based guidelines to maximize cumulative analgesic neuroplasticity while ensuring optimal patient tolerability in the setting of severe central sensitization and hyperalgesia [10,15].

Results

**Figure 1:**
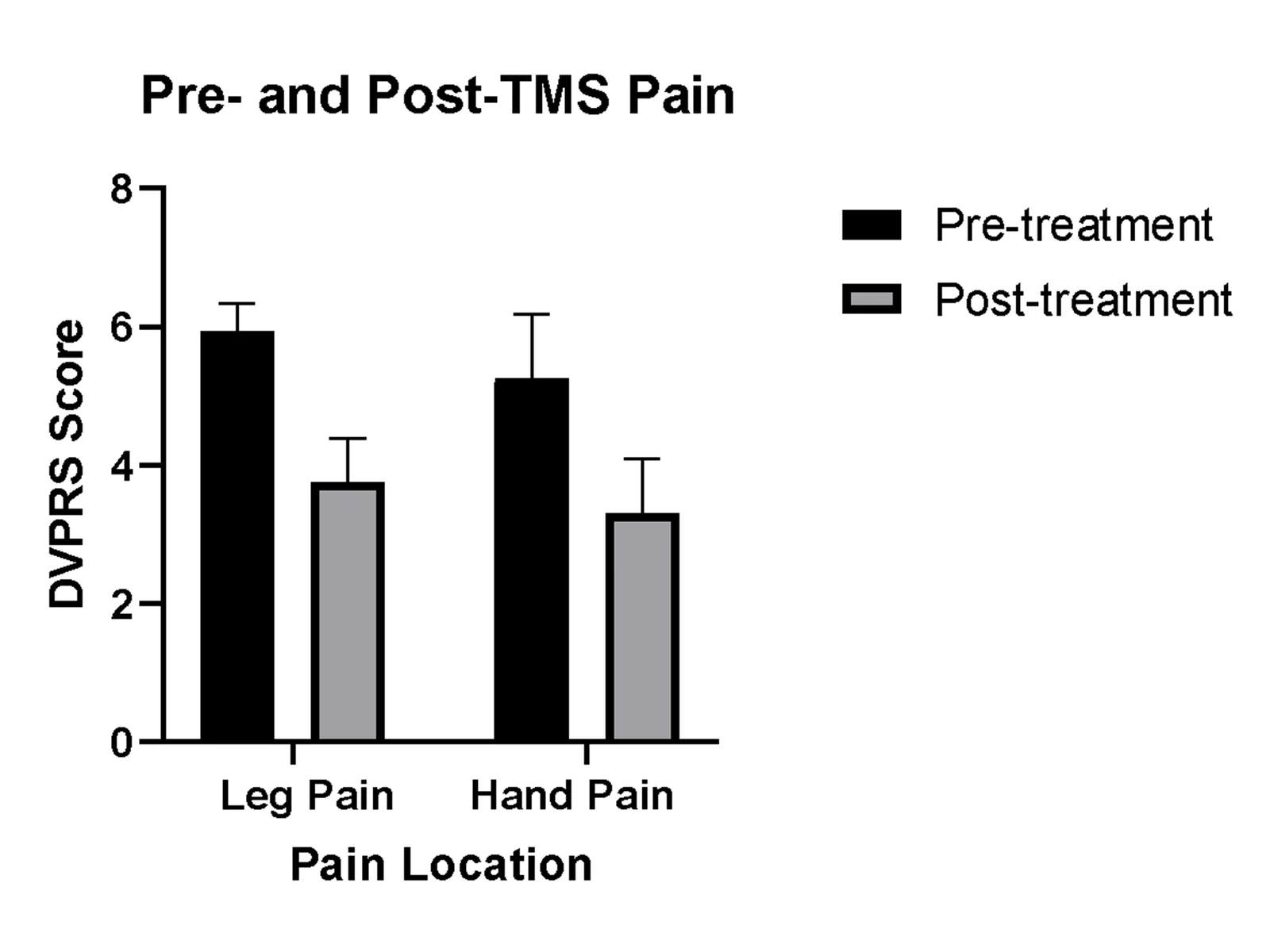
Pre- and post-rTMS pain scores of the legs and hands. Defense and Veterans Pain Rating Scale (DVPRS) current pain scores measured before and after rTMS sessions (21 sessions for leg pain, 14 sessions for hand pain), demonstrating clinically meaningful pain reductions in both the legs and hands. Error bars represent standard deviation.

Descriptive analysis of pre- and post-session data demonstrated clinically meaningful reductions in both hand and leg pain (Figure 1). Pre-session leg pain (mean ± standard deviation) decreased from 5.9 ± 0.41 to 3.8 ± 0.64 following treatment sessions, representing an average pain reduction of 35.6%. Pre-session hand pain decreased from 5.3 ± 0.93 to 3.3 ± 0.80 following treatment sessions, reflecting an average 37.7% reduction. These improvements met established Initiative on Methods, Measurement, and Pain Assessment in Clinical Trials (IMMPACT) benchmarks for clinically meaningful pain relief [16] and aligned with the patient's functional gains and opioid medication tapering over the course of treatment.

The patient reported immediate post-rTMS pain relief that persisted throughout the day, with pain intensity peaking in the morning prior to each session. By the 10th rTMS session, the patient's average daily pain upon awakening was approximately 30% lower than baseline, an improvement that remained consistent through the final session. The patient successfully discontinued tapentadol by the 15th rTMS session under the supervision of his primary care physician.

The patient reported that post-treatment pain was more manageable and less debilitating, enabling a return to daily activities despite intermittent pain, alongside improvements in functional capacity, mood, reduced disability perception, and physical therapy tolerance. The average duration of physical therapy increased from 25 to 45 minutes per session. Increased engagement in non-rehabilitation activities was also observed, such as regular walks with his spouse and child.

Two weeks post-treatment, the patient reported a 15% overall reduction in global pain intensity, with a sustained 10% reduction reported at one-month follow-up, despite confounding physical exertion from a strenuous military relocation.

At one-month follow-up, HRQOL-14 data indicated substantial improvement in mental health indicators. The number of days in the previous month with poor mental health decreased from 25 to 10; days feeling sad, blue, or depressed from 25 to 15; days feeling worried, tense, or anxious from 30 to 10; and days with inadequate rest from 20 to 15.

rTMS was well-tolerated, with no serious adverse events reported. The most common side effect was a post-treatment headache, which occurred after all sessions, although pre-existing headaches improved following rTMS in 43% of sessions. Mild, transient neck or back stiffness was reported in 33% of sessions. During stimulation, the patient experienced mild throbbing discomfort above and behind the left eyebrow but remained awake, comfortable, and conversant throughout.

## Discussion

This case study demonstrates that rTMS targeting the primary motor cortex (M1) was safe, well-tolerated, and associated with clinically meaningful reductions in pain intensity in a patient with multi-limb, treatment-resistant CRPS. Improvements in mood, function, and quality of life facilitated the successful tapering and eventual discontinuation of opioid analgesics, which may reflect the combination of rTMS treatment, physical therapy, and the patient's overall clinical trajectory.

These positive outcomes align with prior research suggesting that rTMS of the motor cortex may modulate multiple analgesic pathways, including the activation of endogenous opioid and dopaminergic systems, inhibition of pain signaling via the corticospinal tract, and enhancement of prefrontal cortical regulation of pain perception [8]. This case demonstrates a favorable clinical response following rTMS in a highly refractory patient and supports the need for further investigation into the therapeutic potential of rTMS for chronic, treatment-resistant neuropathic pain.

The findings from this case are particularly important when considered against the limitations and risks of current therapeutic options for CRPS, including pharmacological treatments and invasive interventions. Opioids, while frequently prescribed, have limited evidence supporting their efficacy in chronic neuropathic pain compared to acute conditions [8,17]. Furthermore, their use in CRPS management is debated due to significant risks, including dependence, overdose, and mortality [17,18]. Other pharmacological treatments are often limited by unfavorable side-effect profiles [19].

Invasive options like spinal cord stimulation have shown some short-term benefit for CRPS Type I, but efficacy often diminishes over time, and the procedure itself carries notable surgical risks (e.g., 3% incidence of significant hemorrhage, 1% mortality rate) [20,21]. Amputation, a last resort, results in permanent disability and does not guarantee pain resolution, with CRPS recurrence reported in some cases [21,22].

In contrast, rTMS therapy is a noninvasive neuromodulation approach associated with a comparatively mild and manageable side-effect profile. Common adverse effects, such as headache and transient neck or back stiffness, are generally short-lived [14,23-25]. The risk of serious adverse events like seizure is rare (estimated at 0.008% per session) and can be substantially mitigated by adhering to safety guidelines, such as using stimulation intensities below 100% of the resting motor threshold [14,23]. Overall, the safety and tolerability data from this case and prior trials [7,10] support rTMS as a well-tolerated and promising therapeutic alternative to polypharmacy and invasive interventions for refractory CRPS.

While the clinical improvements observed in this case are encouraging, several limitations must be noted. First, as a single case study, these findings cannot rule out placebo effects, patient expectations, investigator bias, or natural fluctuations in disease severity over time. Second, outcome assessments were unblinded, and the long-term durability of relief beyond the immediate post-treatment period remains to be characterized. Third, patient-specific variations and concurrent management strategies limit the generalizability of these outcomes to broader CRPS populations. Fourth, specific clinical parameters, including formal Budapest criteria classification and documented nerve injury, could not be incorporated due to institutional compliance restrictions prohibiting chart re-examination following protocol closure. Finally, while rTMS is indicated for major depressive disorder, obsessive-compulsive disorder, and migraine, its application in CRPS represents an off-label clinical use.

To build upon these observational findings, future research should prioritize prospective, double-blind, sham-controlled randomized trials to rigorously evaluate the therapeutic efficacy of rTMS in CRPS. Subsequent investigations should focus on defining optimal stimulation protocols, as well as identifying clinical or neuroimaging biomarkers that may predict favorable treatment response.

## Conclusions

This case study describes a favorable clinical response and good tolerability following rTMS of the primary motor cortex, marked by clinically meaningful pain reduction and improved functional status reported by a patient with refractory, widespread CRPS Type II. While these findings demonstrate feasibility in an individual, highly refractory case, larger controlled studies are required to systematically evaluate the safety, efficacy, and optimal implementation of rTMS in CRPS.
